# Editorial: Expert opinions in genomic analysis

**DOI:** 10.3389/fbinf.2025.1641083

**Published:** 2025-06-19

**Authors:** João C. Setubal, Alberto Paccanaro

**Affiliations:** ^1^ Department of Biochemistry, Institute of Chemistry, University of São Paulo, São Paulo, Brazil; ^2^ School of Applied Mathematics (EMAp), Fundacao Getulio Vargas, Rio de Janeiro, Brazil

**Keywords:** genomics, microbiome, protein design, large language models (LLM), standards, RNA structure prediction, data reuse

Genomics is a term that has over the years expanded its meaning to cover a wide variety of topics, many of which have their roots in genome data but go well beyond them. As an example, in many cases Genomics serves as a stand-in for the more general term genome-based “omics”, such as transcriptomics and proteomics. This Research Topic contains four perspective or opinion papers that touch on a variety of subjects under the general area of Genomics.

Valentini et al. discuss the promises of large language models for protein design and modeling. This perspective piece was published in our Research Topic in November 2023. Almost a year later, in October 2024, one of the Nobel prize winners in Chemistry was David Baker, for his contributions to “computational protein design,” which is excellent evidence for the timely contribution of this article. As the authors argue, the achievements of Large Language Models (LLMs) in natural language processing and the analogies that exist between natural language and the language of proteins make protein design and modeling an excellent application area for LLMs.

von Löhneysen et al. write about the limits of experimental evidence in RNA structure prediction. At first glance, the title is disconcerting: we have all been trained to believe that computational predictions benefit from any experimental evidence. But the authors argue that, although chemical and enzymatic information obtained through experiment can indeed provide essential structural information for RNAs, even with such evidence certain ambiguities about RNA structure remain. Such ambiguities have to be resolved with other methods, particularly comparative analysis.

The Research Topic is wrapped up with two papers on Standards in Genomics, a subject area that is becoming increasingly important, as the number of genomic and metagenomic datasets continues to increase. The first, by Keenum et al., addresses genomic data reuse and reproducibility. The authors provide convincing arguments that common metadata reporting, clear communication, standardized protocols, improved data management infrastructure, ethical guidelines, and collaborative policies that prioritize transparency and accessibility are the keys to enable the scientific community to address the double challenge of reproducibility and data reuse.

The second paper, by Kelliher et al., is a perspective piece that nicely follows up the previous one on the more specific theme of microbiome data reuse. Using results of a survey of more than 700 microbiome researchers, the authors have identified lack of standards in metadata records, challenges with bioinformatics, and problems with data repository submissions as the main reasons that make microbiome data sharing and reuse more difficult than they should be. The authors offer recommendations on how to overcome these challenges.

In sum, we believe this Research Topic to be a nice contribution to a small sample of important and timely topics in genome analysis.

